# Genomic Analysis of Intrinsically Disordered Proteins in the Genus *Camelus*

**DOI:** 10.3390/ijms21114010

**Published:** 2020-06-03

**Authors:** Manal A. Alshehri, Manee M. Manee, Mohamed B. Al-Fageeh, Badr M. Al-Shomrani

**Affiliations:** 1 National Center for Biotechnology, King Abdulaziz City for Science and Technology, Riyadh 11442, Saudi Arabia; Manalalshehri@kacst.edu.sa (M.A.A.); malmanee@kacst.edu.sa (M.M.M.); mfageeh@kacst.edu.sa (M.B.A.-F.); 2 Institute of Bioinformatics, University of Georgia, Athens, GA 30602, USA

**Keywords:** disoredered proteins, *Camelus*, disorder prediction, GO

## Abstract

Intrinsically disordered proteins/regions (IDPs/IDRs) fail to fold completely into 3D structures, but have major roles in determining protein function. While natively disordered proteins/regions have been found to fulfill a wide variety of primary cellular roles, the functions of many disordered proteins in numerous species remain to be uncovered. Here, we perform the first large-scale study of IDPs/IDRs in the genus *Camelus*, one of the most important mammalians in Asia and North Africa, in order to explore the biological roles of these proteins. The study includes the prediction of disordered proteins/regions in *Camelus* species and in humans using multiple state-of-the-art prediction tools. Additionally, we provide a comparative analysis of *Camelus* and *Homo sapiens* IDPs/IDRs for the sake of highlighting the distinctive use of disorder in each genus. Our findings indicate that the human proteome is more disordered than the *Camelus* proteome. Gene Ontology analysis also revealed that *Camelus* IDPs are enriched in glutathione catabolism and lactose biosynthesis.

## 1. Introduction

It has been believed for decades that protein function relies on three-dimensional structure, which is associated with primary sequence [1]. In fact, it has been experimentally proven that some proteins do not fold into a regular structure, or are completely unstructured [2], lacking unique tertiary structures in their native states under physiological conditions. These proteins/regions are called intrinsically disordered proteins/regions (IDPs/IDRs). The absence of three-dimensional structure in these proteins promotes structural flexibility and plasticity, which has been linked with major functional roles [3]. IDPs/IDRs can recognize diverse target molecules with high specificity and low affinity, and many IDPs undergo transitions to more structured states after binding to their partners. For example, molecular recognition features (MoRFs) are disordered regions that undergo a disorder-to-order transition upon participating in protein–protein interactions [4]. Unstructured proteins frequently function as hubs in protein–protein interaction networks; their activities include intracellular signaling cascades, regulation of cellular processes such as translation and transcription [4], involvement in functional sites such as those membranes and nucleic acids, binding to other proteins, cellular localization signals, post-translational modification (PTM), and alternative splicing [5]. Some organisms use the network complexity caused by protein disorder as a simple mechanism for adapting to environmental stress. Specifically, fully disordered proteins are resistant to low temperatures, and they have direct roles in the cold stress tolerance of several species [6]. Furthermore, disordered proteins play roles in many biological processes, including cell cycle control, regulation, and signaling [7], which in turn affect functions related to conditions such as diabetes, cancer, cardiovascular diseases, and neurodegenerative diseases [8,9].

Not only do IDPs play key roles in cellular functions, but they comprise significant proportions of eukaryotic genomes; it has been stated that 52%–67% of eukaryotic proteins are predicted to be disordered [3]. Disordered proteins also comprise 26%–51% and 16%–45% of archaean and bacterial proteins, respectively [3]. Several studies have highlighted that IDPs are predominant in mammalian proteomes [8,10]. In mice, disordered proteins can serve as quantitative probes for biological pathways and processes [11]. In the human genome, 44% of proteins have disordered regions of >30 amino acids [4,7]. IDPs have also been highly associated with several diseases in humans, and disordered proteins may serve as a possible class of targets for drugs that aim to change protein–protein interactions.

Various experimental techniques have been used to detect IDPs/IDRs, including nuclear magnetic resonance (NMR) and small-angle X-ray scattering (SAXS); however, these techniques are time-consuming and expensive to perform [12]. As a result, computational methods for predicting disordered proteins have expanded considerably over the last two decades. Computational approaches can be roughly grouped into three types [4]. The first approach predicts disordered proteins based on sequence properties [13]. The second approach, which is the most widely used, employs machine learning to build predictive models. Examples include DISOPRED2 [7], which uses linear support vector machines (SVMs) trained on known protein datasets, and PONDER XL1 [14], which applies a feed-forward neural network trained on protein sequence features. Finally, methods using the third approach, termed meta-predictors, combine multiple successful predictors with the hope of achieving high prediction accuracy [15,16]. The available databases of experimentally disordered proteins, such as the Database of Protein Disorder (DisProt) [17] and IDEAL [18], provide a wealth of resources for developing and assessing accurate predictors.

This study aimed to predict disordered proteins/regions in *Camelus* species and in humans using multiple state-of-the-art prediction tools. To the best of our knowledge, this is the first study to utilize bioinformatics to predict IDPs and disorder binding regions (DBRs) for the genus *Camelus*, which comprises three species: *Camelus dromedarius*, *Camelus bactrianus*, and *Camelus ferus*. In addition, we provide extensive functional annotation for those proteins in both *Camelus* and *Homo sapiens*, and demonstrate a systematic comparison of IDPs/DBRs and their functional roles in both genera.

## 2. Results

**Figure 1:**
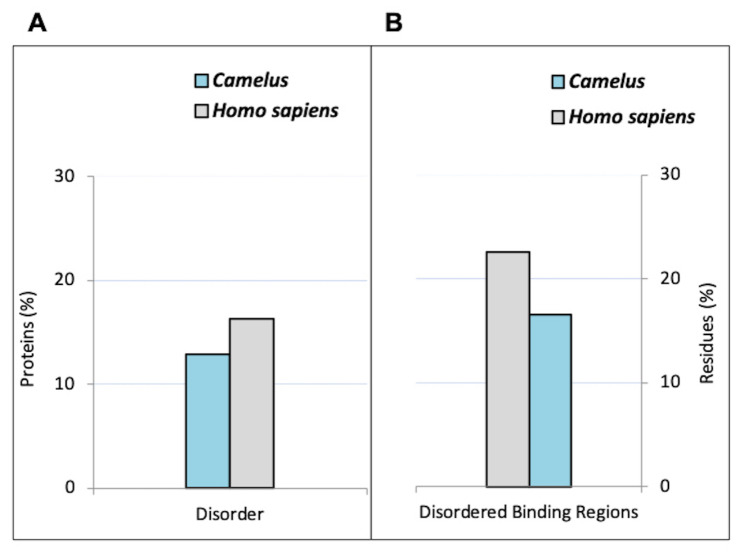
Overall predicted disorder and disorder binding regions (DBRs) in *Camelus* and *Homo sapiens*. (**A**) Percentage of proteins with at least one long disordered region (LDR) with at least 50% disordered residues (according to DISOPRED predictions). (**B**) Percentage of disordered residues involved in binding (according to ANCHOR predictions).

The DISOPRED analysis revealed that the proteome of *Camelus* is less disordered than the human proteome. The mean content of disordered residues in *Camelus* was 28.16%, compared to 34.04% for *Homo sapiens* (Table 1; Chi-square *p*-value < 2.2 ×$10^{-16}$). Furthermore, the percentage of proteins with long disordered regions (LDRs; ≥30 disordered residues) is also higher in the human proteome than in *Camelus*, at 52.56% and 47.16%, respectively (Chi-square *p*-value < 2.2 ×$10^{-16}$). The average length of LDRs is around 170 residues in *Camelus* and 214 residues in *Homo sapiens*, and the mean proportion of residues belonging to LDRs was 17.9% for *Camelus* and 22.88% for *Homo sapiens* (Chi-square *p*-value < 2.2 ×$10^{-16}$). On average, the number of LDRs per protein was 1.14 for *Homo sapiens* vs. 0.95 for *Camelus* (Wilcoxon rank sum *p*-value < 2.2 ×$10^{-16}$). The overall percentage of proteins in which at least half of their content was disordered is illustrated in Figure 1A (12.84% for *Camelus* and 16.31% for *Homo sapiens*). Disorder predictors other than DISOPRED reported similar statistical differences between both genera (Table S1).

**Figure 2:**
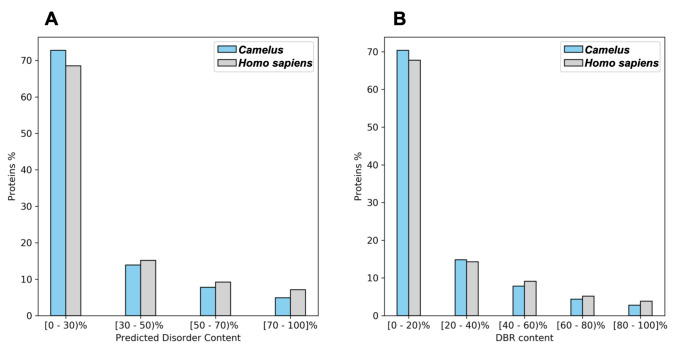
Binning of *Camelus* and *Homo sapiens* proteins by degree of (**A**) predicted disorder (percentage of disordered residues relative to sequence length, predicted by DISOPRED) and (**B**) disorder binding regions (predicted by ANCHOR).

In eukaryotes, disordered proteins are involved in protein–protein interactions. Our bioinformatics analysis showed that 39% of *Homo sapiens’* proteins have at least one disorder binding region that can contribute to such interactions, while the proportion in *Camelus* was 37.75% (Figure 1B; Wilcoxon *p*-value < 2.2 ×$10^{-16}$). Furthermore, the average number of DBRs per protein was slightly higher in *Homo sapiens* than in *Camelus* (0.47 vs. 0.40, Wilcoxon *p*-value < 2.2 ×$10^{-16}$), as was the mean residue content of binding regions (19.69% vs. 16.51%, Wilcoxon *p*-value < 2.2 ×$10^{-16}$). Conversely, the percentage of proteins with less than 40% disordered residues was higher in *Camelus* than in *Homo sapiens* (Figure 2B). By extension, the fraction of proteins with 40% to 100% of their residues in DBRs is larger in *Homo sapiens* than in *Camelus*.

We grouped proteins for each genus by disorder content to visualize the impact of highly disordered proteins (having ≥50% disordered residues). As shown in Figure 2A, we found that proteins having 30% or less disordered residues predominated in both genera, at 72.7% for *Camelus* and 68.5% for *Homo sapiens*. Meanwhile, even though the absolute amount of disordered amino acids represents more than a third of the whole proteome in both genera (Table 1), extremely disordered proteins (having ≥70% disordered residues) represent only 4.9% and 7.1% of the proteome in *Camelus* and *Homo sapiens*, respectively.

### 2.1. Disorder and Conserved Regions

**Figure 3:**
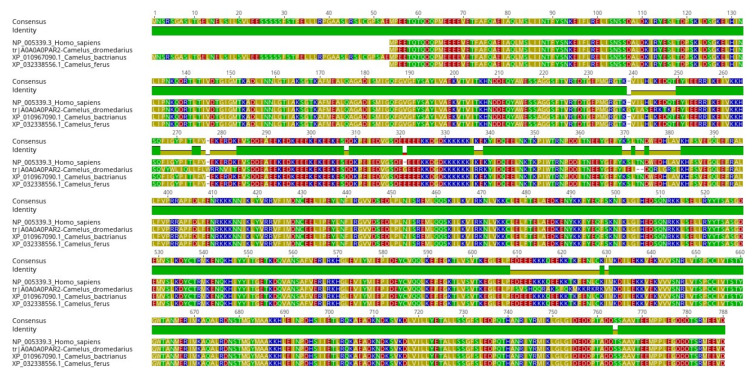
Multiple sequence alignment of four homologous proteins for *Homo sapiens*, *Camelus dromedarius*, *Camelus bactrianus*, and *Camelus ferus*.

**Figure 4:**
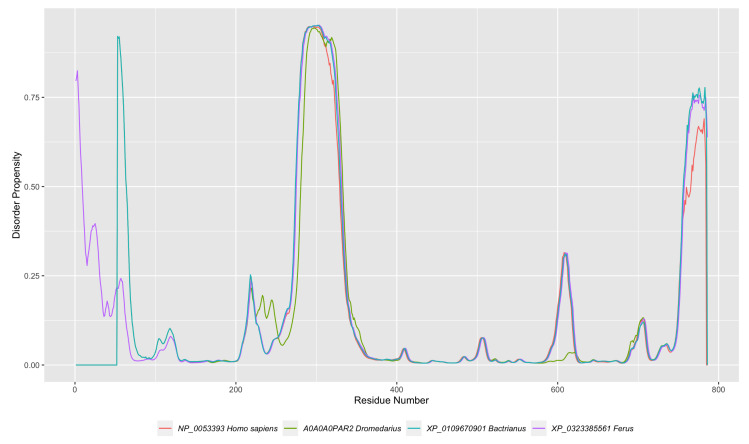
Disorder predispositions of four homologous proteins from *Homo sapiens*, *Camelus dromedarius*, *Camelus bactrianus*, and *Camelus ferus*.

We compared homologous proteins from *Homo sapiens* and the three species in the genus *Camelus* (*Camelus dromedarius*, *Camelus bactrianus*, and *Camelus ferus*). We performed multiple sequence alignment on the four proteins using Geneious Prime software (Figure 3). We identified the disordered regions and then visualized the disorder trend along with each protein (Figure 4). Our findings indicate that among the conserved regions, the disorder is conserved. However, the percentage of disorder prediction might vary slightly. As seen in (Figure 3), the residues from position 761 to the end of the sequences are identical; nevertheless, there is a slight variation in the disorder percentage in this region for humans compared to other proteins (Figure 4).

### 2.2. Functional Annotation

The PANNZER2 server was used to retrieve functional annotations for both protein sets. *Camelus* proteins were annotated with 8739 functional terms from the three Gene Ontology (GO) domains, including 3709 biological process (BP) terms, while *Homo sapiens* proteins were annotated with a total of 12,521 terms, including 4779 BP terms. We predicted 1993 terms in common for *Camelus* and *Homo sapiens* proteins, which were used for the comparative analysis of the two groups.

### 2.3. Gene Ontology Enrichment Analysis

Two enrichment analyses were performed, the first identifying GO functional classes significantly enriched in the disordered proteins of *Camelus*, and the second identifying GO functional classes that were distinctively related to disordered proteins in *Camelus* compared to *Homo sapiens*. A given GO term can show up in the first analysis (disordered in *Camelus*) but not in the second (comparison with *Homo sapiens*) when the amount of disorder is similar in both organisms. In contrast, the presence of a term in the results of the second analysis but not the first indicates that, while the disorder content of that functional class is not remarkably high in *Camelus*, it is still noticeably higher than in *Homo sapiens*. Finally, a term showing up in both analyses would be both significantly enriched in disorder in *Camelus* and more disordered in that genus than in *Homo sapiens*. The list of GO terms for both analyses is available (Table S2). In the following sections, we discuss in detail the Gene Ontology enrichments identified by each analysis.

### 2.4. Functional Categories Significantly Disordered in Camelus

**Figure 5:**
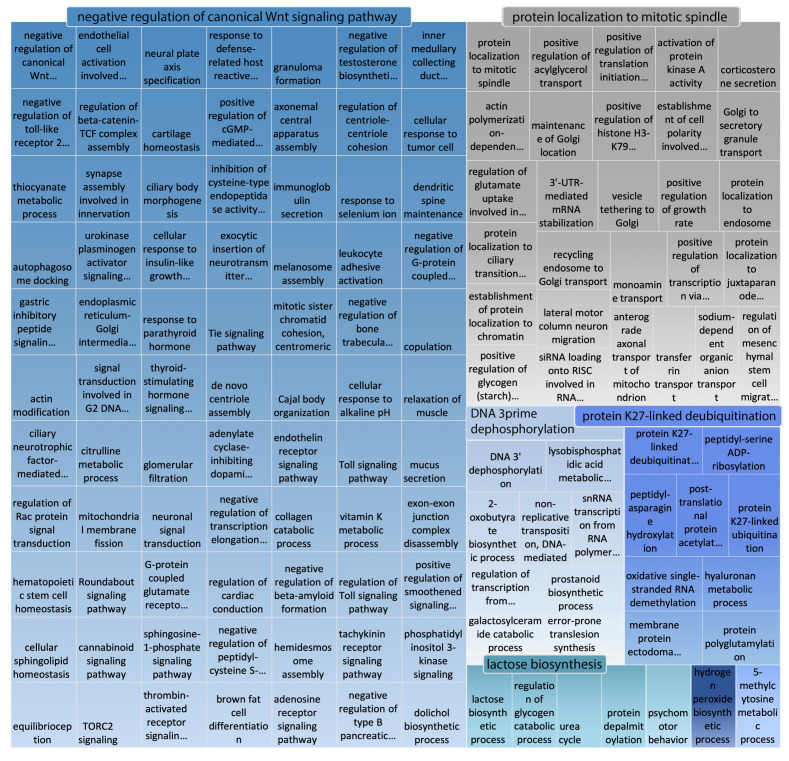
REVIGO representation of GO biological process terms (with PPV > 0.7) that are significantly enriched in disordered *Camelus* proteins. Disordered proteins are those containing at least one “long disordered region” based on DISOPRED predictions.

The main biological process (BP) categories enriched in disordered *Camelus* proteins (proteins having at least one LDW based on DISOPRED predictions) are illustrated in Figure 5. As PANZZER reported vast lists of GO terms, we used REVIGO to assist in their functional interpretation by performing statistical analysis on the most significant GO terms and depicting those functions as a treemap (Figure 5). There were 130 GO terms enriched in *Camelus* disordered proteins, of which the vast majority were children of the term “negative regulation of canonical Wnt signaling pathway involved in osteoblast differentiation”. Within this category, most enriched terms were related to signaling pathways. The second-largest functional cluster impacted by disorder in *Camelus* was “protein localization to mitotic spindle”; that category could be summarized as “localization and transport”. Other prominently enriched terms were “protein K27-linked deubiquitination”, “DNA 3prime dephosphorylation”, and “lactose biosynthesis”. The complete list of 130 terms is available (Table S2).

### 2.5. Comparison of GO Functional Categories between Camelus and Homo sapiens

**Figure 6:**
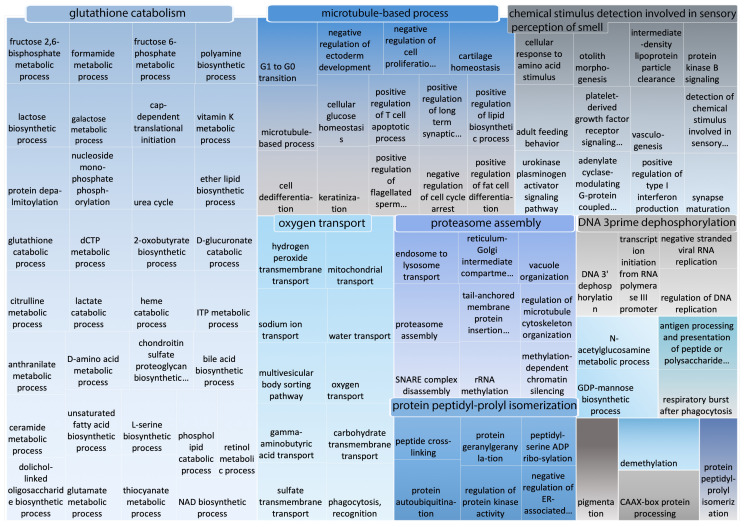
REVIGO representation of GO biological process terms (with PPV > 0.7) that are more enriched in *Camelus* disordered proteins than in those of *Homo sapiens*. Disordered proteins are those containing at least one “long disordered region” based on DISOPRED predictions.

Figure 6 shows BP functional categories that were significantly overrepresented among *Camelus* disordered proteins when compared to *Homo sapiens*. Of the 130 terms significantly enriched in *Camelus* disordered proteins overall, REVIGO reported only 96 to be more disordered in this genus than in *Homo sapiens*. The complete list of those terms is available in Table S2. The most highly represented categories were “glutathione catabolism”, “microtubule-based process”, “detection of chemical stimulus involved in sensory perception of smell”, and “oxygen transport” (Figure 6), of which the single largest functional cluster was “glutathione catabolism”. This category contains several catabolic processes, including “lactate catabolic process”, “heme catabolic process”, and “phospholipid catabolic process”.

The disappearance of some terms, such as “hydrogen peroxide biosynthesis” and “psychomotor behavior”, from the results of this second analysis indicates that the disorder content of these particular categories is either similar in *Homo sapiens* and *Camelus*, or is higher in *Homo sapiens*. Conversely, the category of “oxygen transport” (including hydrogen peroxide transmembrane transport, sodium ion transport, and water transport) was enriched only in the second analysis, indicating that these processes were more disordered in *Camelus* than in *Homo sapiens*. Meanwhile, as seen in Figure 5 and Figure 6, the function “DNA 3prime dephosphorylation” appeared in the results of both analyses, and thus is one of the main functions that are significantly disordered in *Camelus* while also being more disordered relative to the human proteome. The complete sets of GO parent terms that are enriched in *Camelus* disordered proteins and more enriched in *Camelus* disordered proteins than in *Homo sapiens* are given in Table 2.

**Figure 7:**
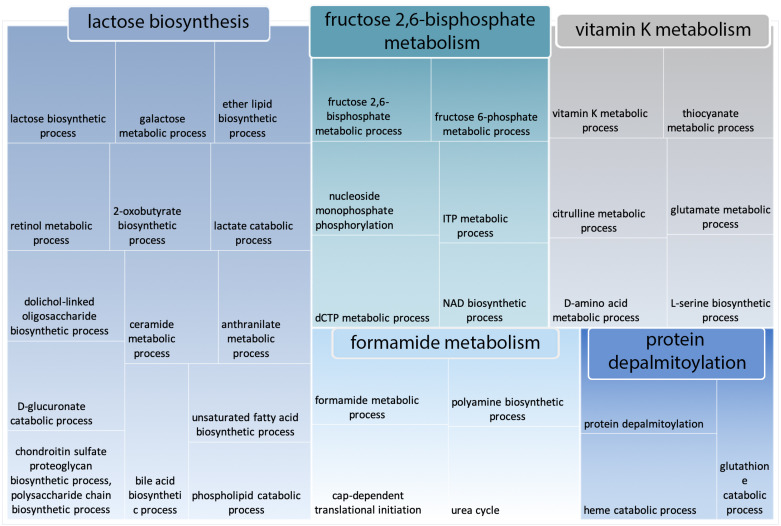
REVIGO representation of all GO terms included within the category “glutathione catabolism”, which comprises the largest cluster of GO terms that are more enriched in *Camelus* disordered proteins compared to those of *Homo sapiens*.

To gain deeper insight into the largest category “glutathione catabolism” (Figure 6), we passed the terms under this category into REVIGO for clustering into more meaningful sub-groups. Surprisingly, the category “lactose biosynthesis”, which was significantly enriched in *Camelus* disordered proteins, appeared again in this analysis as being noticeably more enriched in the *Camelus* proteome than in that of *Homo sapiens* (Figure 7).

## 3. Discussion

In contrast to the traditional perspective that associates protein function with 3D structure, IDPs and IDRs are highly prevalent in many genomes, and they play vital functional roles in diverse cellular processes. In particular, the capability of disordered proteins to be involved in one-to-many interactions is one of the tricks organisms use to increase protein network complexity without expanding the network size [3]. Accordingly, whole-genome studies have reported that the proportion of disordered proteins increases with increasing complexity of an organism [7,19]. In our study, we found that the human proteome is more disordered than that of the genus *Camelus*. This trend is maintained across different disorder predictors and the use of different criteria for identifying disorder.

In eukaryotic cells, IDPs/IDRs are essential mediators of the control of signaling machinery and post-translational modifications [20,21]. In agreement with previous studies that have emphasized the prevalence of signaling and regulation functions among disordered proteins [7,8,9,10], we observed that pathway signaling and regulation were the most enriched functions among disordered *Camelus* proteins. Furthermore, this function was not overrepresented when comparing the *Camelus* proteome with that of *Homo sapiens*, which is attributable to it being a prevalent function of IDPs in all eukaryotes [3,7]. These processes are commonly more complex in eukaryotes than in prokaryotes and have been previously related to disorder in higher organisms. In contrast, we found that dephosphorylation processes are not only extensively enriched in *Camelus* disordered proteins but also more enriched relative to *Homo sapiens*. Other functional terms for which the disorder level was significantly higher in *Camelus* than in *Homo sapiens* included “microtubule-based process”, “proteasome assembly”, and “oxygen transport”.

Our findings showed that the synthesis of lactose in *Camelus* is dominated by disordered proteins. Moreover, the major biological functions that were more enriched in *Camelus* disordered proteins relative to those of humans were “lactose biosynthesis” and “glutathione catabolism”. The characteristic example is α-Lactalbumin protein, which is a disordered protein in camels [22]. This protein is known to be involved in catalyzing the last step in lactose biosynthesis.

The systematic comparative in this study shows that despite *Homo sapiens’* proteome being more disordered than that of the genus *Camelus*, there are some GO functional classes are significantly enriched in disordered proteins in the genus *Camelus* when compared to humans. This work may provide worthy information for understanding the organism complexity when considering IDPs.

IDPs or IDRs occupy a fraction of the *Camelus* proteome. Authors in [23] revealed that the C-terminal is more disordered than the N-terminal in the cLin-28 protein of *Camelus dromedarius*. Our future work will extend this study to show sequence compositions of IDPs residues and analyze their occurrence in *Camelus* proteins. Furthermore, it is worth understanding the effects of IDRs contents on the protein structure. Therefore, more efforts are required to investigate the role of MoRFs and their interactions with other partners, and their effects on the protein function in the genus *Camelus*.

In conclusion, in the genus *Camelus*, the proportion of disordered proteins is considerable in functions such as regulation of signaling and dephosphorylation. This outcome is in line with what has been published for disordered proteins in other organisms. However, when compared to *Homo sapiens’* proteome, the *Camelus* proteome is also particularly enriched in disordered proteins for other important functions, such as lactose biosynthesis and oxygen transport. Our findings suggest that synthesis of one of the critical components of camel milk, lactose, is not only significantly enriched in disordered proteins, but that the level of disorder in this biological function for the genus *Camelus* is remarkably high as well. However, more studies are needed to understand the role of disordered proteins in lactose synthesis and how the unique characteristics of disordered proteins affect the quality of camel milk.

## 4. Materials and Methods

### 4.1. Protein Dataset

We collected protein sequences of *Homo sapiens* and the genus *Camelus* from the Protein Knowledgebase (UniProtKB, release 2019, https://www.uniprot.org). We searched for *Homo sapiens* proteins directly using the UniProtKB search engine, which yielded 73,947 canonical proteins and isoforms. To download the proteome of the *Camelus* genus, we selected the “organism” option in UniProtKB and searched using as keywords the three *Camelus* species (*Camelus dromedarius*, *Camelus bactrianus*, and *Camelus ferus*), which returned 20,745 canonical proteins and isoforms. We removed redundant sequences by clustering similar proteins using the CD-HIT tool (threshold = 60%) [24]. The dataset was also cleaned by filtering out sequences containing ambiguous residues (e.g., B, X, Z). The final dataset contained 22,156 and 18,338 proteins for *Homo sapiens* and *Camelus*, respectively.

### 4.2. Protein Disorder Prediction

Prediction of disordered proteins was implemented using three different tools: DISOPRED v3.1 [25], IUPred [26], and ESpritz [27]. DISOPRED 3 is based on a support vector machine (SVM), neural network, and nearest neighbor classifier; IUPred relies on an energy estimation approach; and ESpritz developed using bidirectional neural network algorithm. All three tools accept one protein as input, and generate for each amino acid in its sequence a disorder probability in the range (0.0–1.0). Residues with values 0.5 or higher are predicted to be disordered.

We analyzed predicted disordered proteins in three different respects. Firstly, we computed the percentage of disordered residues in each protein for both datasets (*Camelus* and *Homo sapiens*). We also identified segments that were at least 30 consecutive disordered residues long, termed long disordered regions (LDRs). Finally, we detected disordered regions involved in protein–protein interactions, termed disorder binding regions (DBRs), using ANCHOR (based on IUPred) [28]. Similarly to disorder predictors, ANCHOR gives a score of 0.5 or above for disordered amino acids. For a region to be considered a DBR, it needed to contain at least 30 disordered residues.

### 4.3. Multiple Sequence Alignment

The multiple sequence alignment between the four proteins from *Homo sapiens* and the three organisms of the genus *Camelus* (*Camelus dromedarius*, *Camelus bactrianus*, and *Camelus ferus*) with accession numbers (NP_005339, A0A0A0PAR2, XP_010967090 and XP_032338556) was performed using Geneious Prime software v11.0.6 [29].

### 4.4. Functional Annotation

We used Gene Ontology (GO) terms defined by the Gene Ontology Consortium to associate functional terms with protein sequences. GO terms describe different functional roles of genes and gene products, and are grouped into three domains (sub-ontologies): biological processes, cellular components, and molecular functions. The GO annotations of our dataset were predicted using Protein ANNotation with Z-scoRE (PANNZER2) [30], which provides functional annotations for proteins with unknown functions by searching for homologous proteins in the Uniprot database. The scoring function selected was ARGOT [31], and the scientific names of species were subsequently adjusted to *Camelus* and *Homo sapiens*. We considered a GO term to be associated with a protein if it had an estimated positive predictive value (PPV) above 0.7. The protein sequences for GO annotation were submitted on December 29, 2019 (PANNZER2 databases are updated monthly).

### 4.5. Gene Ontology Enrichment Analysis

To evaluate the association of protein disorder with Gene Ontology (GO) classes, we performed two analyses. A protein was considered disordered if it contained at least one “long disordered region (LDR)” according to DISOPRED predictions. In the first analysis, we evaluated those GO classes significantly enriched in disordered proteins in *Camelus*. In the second analysis, we evaluated functional classes differentially enriched in disordered proteins in *Camelus* when compared to *Homo sapiens*. In our study, we focused on biological functions that were more disordered in *Camelus* than in humans.

**Figure 8:**
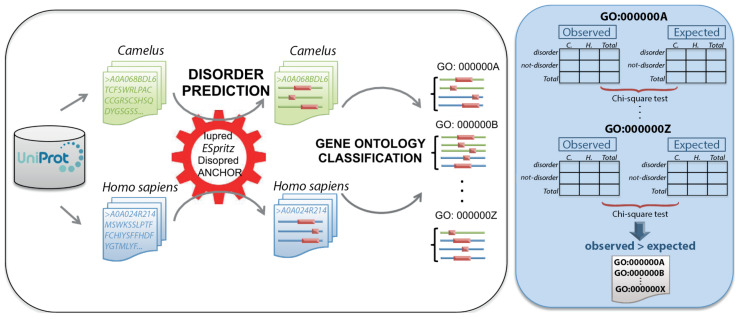
Schematic representation of the methodology used for the comparative study of disordered proteins in *Camelus* (green) and *Homo sapiens* (blue). For each organism, protein sequences were retrieved from Uniprot. For each protein, disordered regions (pink) were predicted using three different methods (Iupred, ESpritz, and DISOPRED), and disordered binding regions (DBRs) were predicted using ANCHOR. Disordered proteins were then assigned to GO:BP functional classes using PANZZER, and a comparative analysis of disorder levels between the two genera was performed for each class. Contingency tables were constructed with the per-genus counts of disordered and not-disordered proteins and a Chi-squared test applied. GO classes for which the difference in disorder was positive for *Camelus* were considered to be more disordered in *Camelus* than in *Homo sapiens*.

To perform a comparative analysis of functional classes common to disordered proteins in *Camelus* and humans, we applied the method described in (Figure 8), which was consistent with previous studies [19,32]. First, we created a contingency table (2 × 2) for each GO term to analyze the association between two categorical variables: protein status (disordered/not-disordered, in rows) and species (*Camelus*/*Homo sapiens*, in columns). Table 3 displays an example for the term GO:0050911. We used Pearson’s Chi-square test to evaluate the significance of the difference between observed and expected counts of disordered proteins in *Camelus* and *Homo sapiens*. We considered only those GO terms for which the difference in disorder was positive for *Camelus*. Using this process, we created a probability value (*p*-value) for each GO class common to *Camelus* and *Homo sapiens*. The smaller the *p*-value, the more enriched the corresponding GO class in *Camelus* relative to *Homo sapiens*. We additionally computed the average number of disordered proteins in each genus for each GO class.

The set of enriched GO terms returned by each analysis available in (Table S2) was used as input for the REVIGO tool [33], which takes long lists of GO terms and clusters them based on semantic similarity in order to remove functional redundancies. The server outputs a reduced set of representative terms that are easier to visualize and interpret (Figure 5 and Figure 6). The set of non-redundant GO terms was depicted as a treemap, which consists of representative umbrella terms (headings in rounded rectangles) that contain several superclusters. Cluster representatives are given a broader name that symbolizes a general function common to all included superclusters, and each supercluster represents a generic function common to all integrated GO classes.

In our analyses, there are three possible outcomes for a given GO term. Firstly, a GO term appearing in the results of the first analysis but not the second either has similar disorder distribution in both genera, or might have greater disorder in *Homo sapiens*. Secondly, a term showing up only in the second analysis indicates that although the disorder content of that functional term is not remarkably high in *Camelus*, it is nonetheless higher than in *Homo sapiens*. Finally, the appearance of a GO term in both tests indicates a class that is significantly disordered in the *Camelus* proteome and also more enriched in disordered proteins for *Camelus* than for *Homo sapiens*.

All statistical analyses and data processing were implemented using the programming languages Python, Perl, and R.

## Figures and Tables

**Table 1:** Summary of disorder prediction by DISOPRED and disorder binding region prediction by ANCHOR in *Camelus* and *Homo sapiens*.

	*Camelus*	*Homo sapiens*
Mean content of disordered residues	28.16%	34.04%
Proteins with at least one LDR	47.16%	52.56%
Mean number of residues belonging to LDR	17.9%	22.88%
Mean number of LDRs	0.95	1.14
Proteins with at least one DBR	37.75%	39.58%
Mean DBRs per protein	0.40	0.47
Mean residues belonging to DBRs	16.51%	19.69%

**Table 2:** Representative functional terms enriched in disordered *Camelus* proteins and those more enriched in *Camelus* relative to *Homo sapiens*.

GO Terms Significantly Enriched in *Camelus* Disordered Proteins	GO Terms More Enriched in *Camelus*
	Than *Homo sapiens* Disordered Proteins
	- glutathione catabolism
	- microtubule-based process
- negative regulation of canonical Wnt signaling	- detection of chemical stimulus
pathway involved in osteoblast differentiation	- involved in sensory perception of smell
- protein localization to mitotic spindle	- oxygen transport
- protein K27linked deubiquitination	- proteasome assembly
- lactose biosynthesis	- protein peptidylprolyl isomerization
- hydrogen peroxide biosynthesis	- N-acetylglucosamine metabolism
- psychomotor behavior	- antigen processing and presentation of peptide
- 5methylcytosine metabolism	or polysaccharide antigen via MHC class II
	- pigmentation
	- demethylation
DNA 3prime dephosphorylation	

**Table 3:** Representative contingency table (2 × 2) constructed for all GO terms common to *Camelus* and *Homo sapiens*, in this case for term GO:00509011.

	*Camelus*	*Homo sapiens*	Total
Disordered	111	2	113
Not-disordered	211	209	420
Total	322	211	533

## Supplementary Materials

The following are available at https://www.mdpi.com/1422-0067/21/11/4010/s1.
